# Supplementary material for: Translational contributions to tissue specificity in rhythmic and constitutive gene expression
Source: Genome Biol. 2017 Jun 16;18:116. doi: 10.1186/s13059-017-1222-2 (PMC5473967; doi:10.1186/s13059-017-1222-2)
Supplement: Supplementary file 6 — Transcriptome-wide kidney RPF (blue) and RNA (orange) levels in the left panels (with “error bars” connecting the two replicates of each timepoint) and TE in the right panels. (ZIP 116896 kb) [file 13059_2017_1222_MOESM6_ESM.zip › Supp_Dataset_S1/A_RNA_non_rhythmic_RPF_non_rhythmic/Banf1_kidney_set_A.pdf]

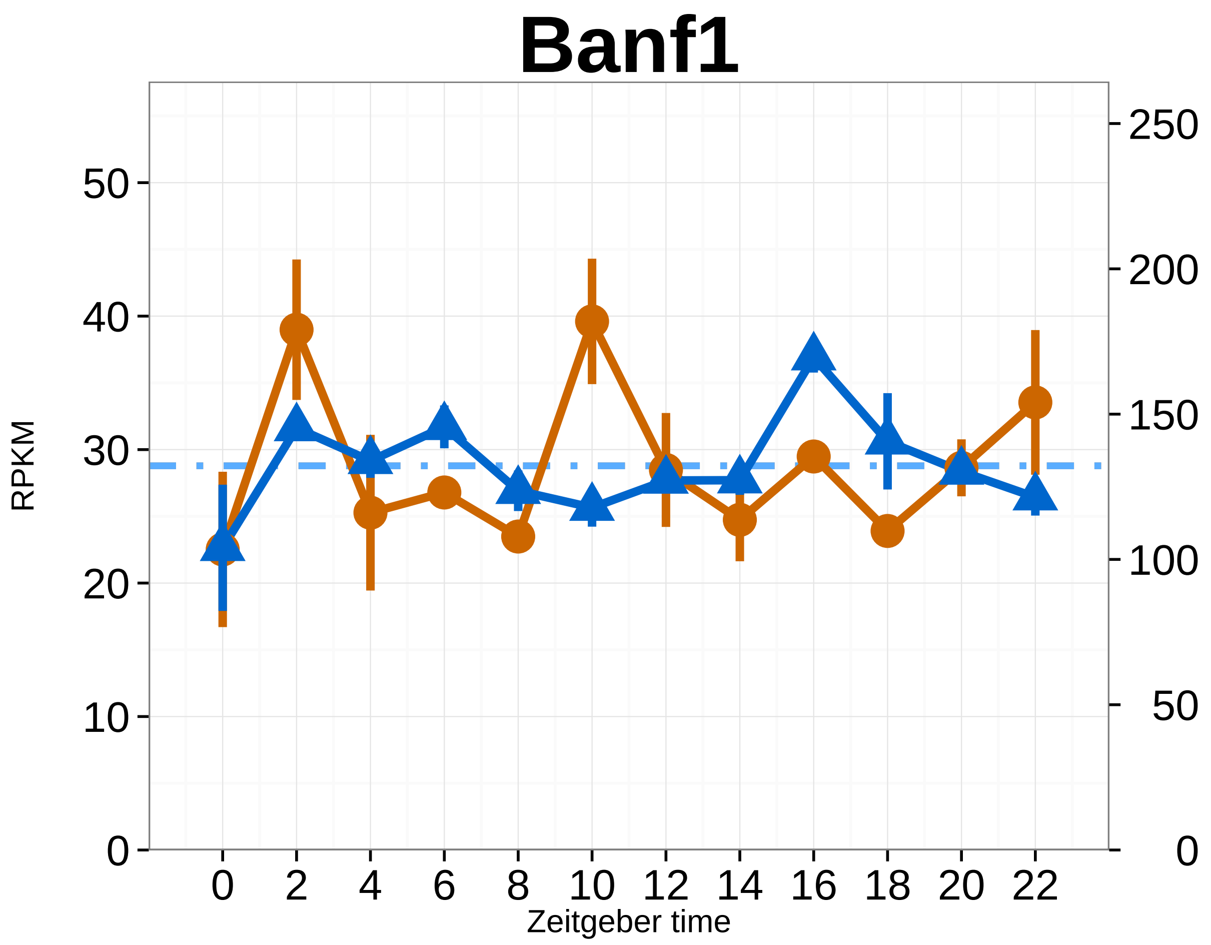
Banf1
RPKM
Zeitgeber time
0
10
20
30
40
50
0
50
100
150
200
250
0 2 4 6 8 10 12 14 16 18 20 22

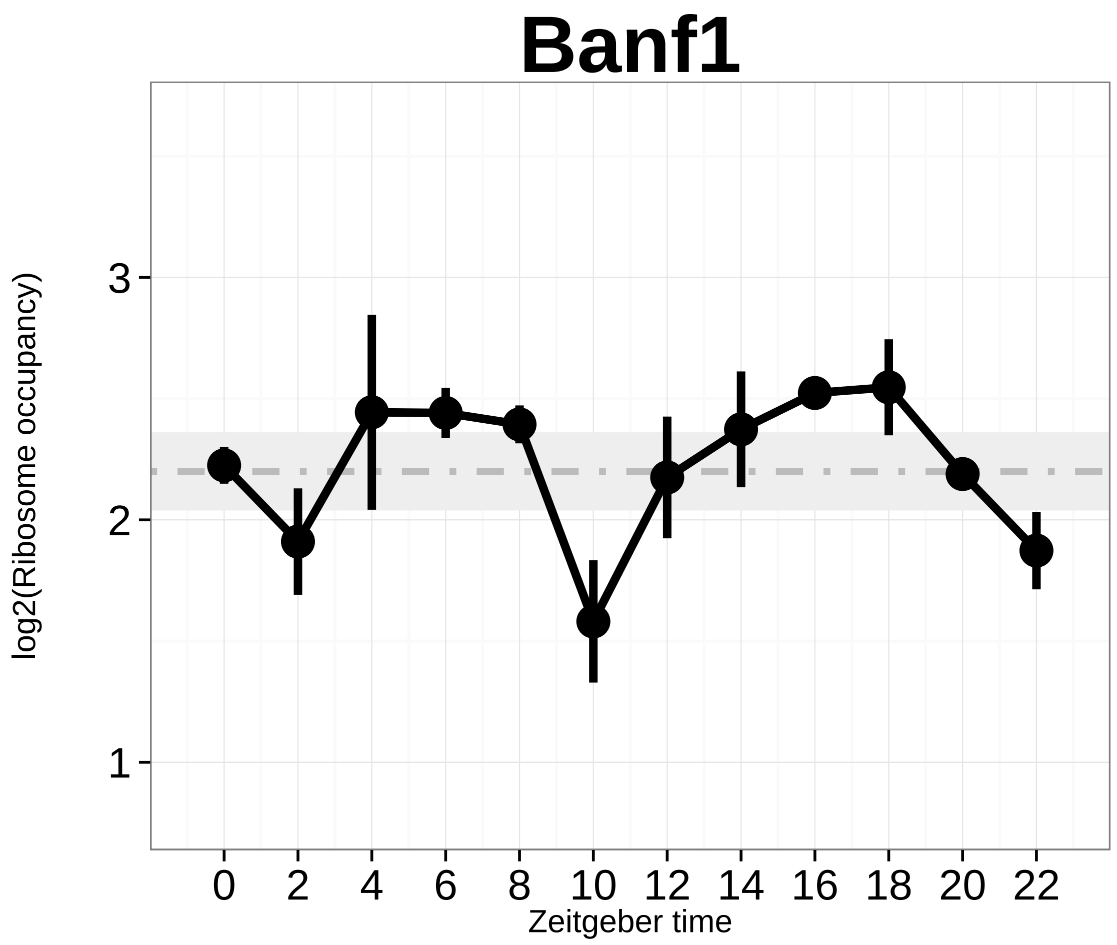
Banf1
log2(Ribosome occupancy)
Zeitgeber time
1
2
3
0 2 4 6 8 10 12 14 16 18 20 22
